# Response to: Use of prior odds for missing persons identifications - authors' reply

**DOI:** 10.1186/2041-2223-3-3

**Published:** 2012-02-01

**Authors:** Bruce Budowle, Jianye Ge, Ranajit Chakraborty, Harrell Gill-King

**Affiliations:** 1 Institute of Applied Genetics, University of North Texas Health Science Center, Fort Worth, TX, USA; 2 Department of Forensic and Investigative Genetics, University of North Texas Health Science Center, Fort Worth, TX, USA; 3 Department of Biological Sciences, University of North Texas, Denton, TX, USA

**Keywords:** Guidelines, human identity, legal proceedings, prior odds, subjectivity

## Abstract

Please see related article: http://www.investigativegenetics.com/content/3/1/2

## Letter to the Editor

We are concerned that statisticians, such as Biedermann *et al. *[1], advocate the position that data may not be needed to support assumptions 'as long as probability is properly considered as an expression of personal belief'. At a time when the National Academy of Sciences [2] has urged the need for the forensic science community to provide reliable results based on 'objective' data, these authors' position cannot be reconciled. The Report noted (on its page 8), 'The simple reality is that the interpretation of forensic evidence is not always based on scientific studies to determine its validity ... A body of research is required to establish the limits and measures of performance and to address the impact of sources of variability and potential bias. Such research is sorely needed, but it seems to be lacking in most of the forensic disciplines that rely on subjective assessments of matching characteristics. These disciplines need to develop rigorous protocols to guide these subjective interpretations and pursue equally rigorous research and evaluation programs'. It is this approach that distinguishes science from other epistemologies. Then the Report called for research in its Recommendation 3 (page 23), 'Research is needed to address issues of accuracy, reliability, and validity in the forensic science disciplines ... [and in section c of Recommendation 3] the development of quantifiable measures of uncertainty in the conclusions of forensic analyses'.

Foremost, none should abide the inclusion of overstated evidence in reports or legal proceedings as it can impinge on the presumption of innocence. The tenet of this presumption should be held dearly by all, and we as scientists should strive to reduce practices that cannot be supported. Biedermann *et al. *[1] appear to argue that, because there is 'subjectivity in science', one does not necessarily have to be held to a standard of justifying assumptions. It is well accepted that there is subjectivity in science. Indeed, Budowle *et al. *[3] and others (see references in [3]) have discussed the inherent subjectivity that exists. It may never be possible to remove all subjectivity, and for that reason we should attempt to develop and agree upon guidelines (or rules) about methods to minimize faulty generalizations. The assertion of Biedermann *et al. *[1] that we do 'not require new guidelines edited by the forensic DNA community' is a formula for continued misapplication. Scientists endeavor to identify the subjectivities and the consequences that might shape or misdirect our conclusions. Our use of the term objectivity [4] is clear in context. Data should support the prior probabilities that are employed. In this regard, we maintain our position.

Budowle *et al. *[4] described the Collins case [5], which held that the courts are concerned about the assumptions made by scientists being justified with actual data that support them. Oddly, Biedermann *et al*. stated 'it remains questionable whether scientists should interfere with a topic of which practicing legal decision makers are already well aware and that, above all, they are in a better position to appreciate' and cite the case State of New Jersey versus Spann [6]. Biedermann *et al*. have misunderstood the decisions in the Spann and Collins cases. These cases fully support our call for an empirical basis for assumptions and that untethered use of personal belief is not acceptable. The court is calling for more justification and guidance and that scientists not usurp the responsibility of the jury. Courts typically do not prescribe science to scientists; they demand that the scientists support their conclusions with data. Personal beliefs alone are not a sufficient basis for entering scientific evidence into legal proceedings. However, in contrast to the misunderstandings of Biedermann *et al*., the Courts in Collins and Spann have prescribed what is not scientifically acceptable.

Contrary to the position promoted by Biedermann *et al. *[1], we advocate that conclusions by scientists be supported with data. For too long the human identity community has avoided addressing the issue of generating prior probabilities in missing persons identification cases. Indeed, in a number of cases, inflated values have been proffered. We reiterate that none of us should abide overstated evidence being presented in legal proceedings or being placed in reports. Our position remains that guidelines are needed so that the triers of fact can use better informed judgment. At the least the legal community can be better educated on the pitfalls wherein scientists may become trapped to ensure the presumption of innocence is protected and justice is served.

It is worth noting that Jackson *et al. *[7], of which one of the authors is prominently cited by Biedermann *et al. *[1] to support their opinion in their letter, opined that 'This information and knowledge may provide a valid, robust assessment of the priors but there is a risk of the scientist being swayed by unreliable or limited prior information, from witnesses for example, towards an erroneous high prior for one hypothesis' and 'We believe that any expression of posterior probabilities, without making clear the priors, and the information on which the priors were based, runs a serious risk of providing misleading opinion'. Perhaps Biedermann *et al*. missed these concerns when reviewing the literature.

## Competing interests

The authors declare that they have no competing interests.

## Authors' contributions

BB was responsible for drafting the response. JG, RC, and HG-K contributed to the response and provided edits. All authors read and approved the final reply.
